# Bilateral Facial Nerve Palsy: A Rare Complication of Chickenpox

**DOI:** 10.7759/cureus.96153

**Published:** 2025-11-05

**Authors:** Bhargavi Bachhu, N. Senthil, Suja Lakshmanan, Vaasanthi R

**Affiliations:** 1 General Medicine, Sri Ramachandra Institute of Higher Education and Research, Chennai, IND; 2 Internal Medicine, Sri Ramachandra Institute of Higher Education and Research, Chennai, IND

**Keywords:** bilateral facial nerve palsy, chickenpox complication, facial nerve paralysis, immunocompetent adult, neurological complication, varicella zoster (chicken pox)

## Abstract

We report a rare case of bilateral lower motor neuron facial nerve palsy in an immunocompetent woman in her 50s following varicella infection. She presented with complaints of difficulty in closing both eyes, difficulty chewing food, deviation of the oral commissure, more pronounced on the right side, and loss of taste sensation one month after chickenpox infection. Examination revealed bilateral lower motor neuron facial weakness along with a few crusted vesicles over the face and extremities, suggesting varicella-zoster virus (VZV) infection. The diagnosis of varicella was made clinically based on the characteristic vesicular rash and illness course. Clinical evaluation and investigations ruled out other alternative etiologies. She responded well to oral corticosteroids and physiotherapy, achieving full recovery within one month. This case highlights an uncommon neurological complication of varicella and emphasizes the importance of vaccination in preventing such complications.

## Introduction

Chickenpox is caused by infection with the varicella-zoster virus (VZV), a highly contagious disease. The World Health Organization estimates the annual global burden of varicella to be approximately 140 million cases [1]. It can occasionally lead to complications. Neurological complications are rare, occurring in only 0.01% to 0.03% of cases [2]; they commonly include meningitis, encephalitis, acute cerebellar ataxia, and transverse myelitis. Isolated facial nerve paralysis is an extremely rare complication of varicella. In a previous study done by Shiihara et al., facial nerve palsy was reported in 8.3% of the patients with neurological complications, indicating that it is an uncommon manifestation of varicella [3]. It can have unilateral or bilateral involvement. It may take five days before or 16 days after the appearance of vesicles for the development of facial nerve palsy [4]. Antiviral therapy is the mainstay of treatment, and corticosteroids, along with physiotherapy, can also be beneficial. However, recognition of bilateral facial nerve palsy as a manifestation of chickenpox remains challenging because of its rarity and its overlap with other causes of facial nerve palsy. We report a rare case of bilateral facial nerve palsy following chickenpox in an immunocompetent woman in her fifth decade of life.

## Case presentation

A woman in her 50s, with no prior comorbidities, presented with a history of difficulty in closing both eyes, more pronounced in the left eye. She also experienced difficulty in eating, drooling from both sides of the mouth. She also had a deviation of the oral commissure, more pronounced on the right side, and a loss of taste sensation for one week. There was no history of headache, vomiting, fever, loss of consciousness, seizures, altered sensorium, or weakness; she also denied a history of retroauricular pain, hearing loss, or vertigo. There was also no history of cough or shortness of breath.

**Figure 1:**
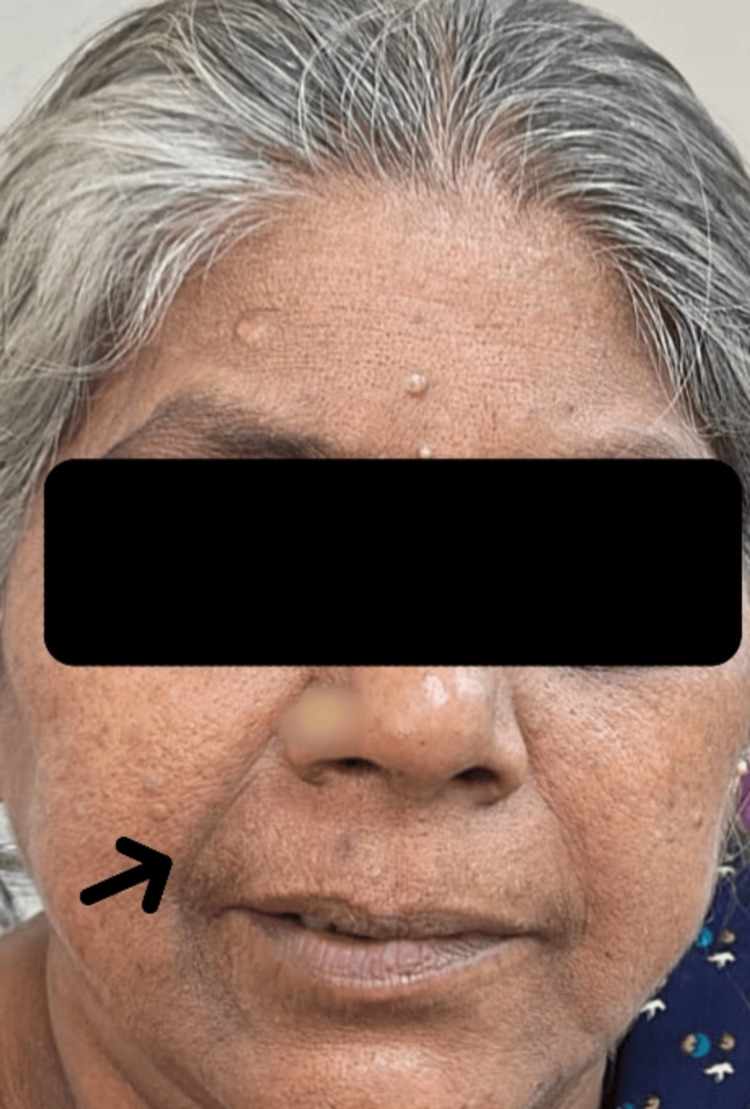
Clinical photograph of the elderly female patient demonstrating bilateral lower motor neuron facial nerve palsy The image shows loss of forehead wrinkling, flattening of bilateral nasolabial folds, and deviation of the oral commissure, which is more prominent on the right side. Note: The patient consented to the use of her images in an open-access journal, and a written and signed consent statement from the patient was provided to the journal.

On further probing, she reported a history of chickenpox infection one month prior. This began with fever, which was followed by the development of multiple vesicles on the face, extremities, and trunk, which later ruptured. She was treated at an outside hospital with 800 mg of acyclovir for seven days and was symptomatically better until the occurrence of these new symptoms. On examination, the patient was conscious and oriented. Her pulse rate was 84 beats per minute, and her blood pressure was 130/80 mmHg. General examination revealed a few dry, crusted vesicles on the face, extremities, and trunk. A palpable lymph node was present in the left supraclavicular region. Cranial nerve examination revealed bilateral lower motor neuron facial palsy, graded as House-Brackmann grade IV (Figure 1). Other neurological examinations revealed normal motor and sensory function, as well as normal cerebellar function. There were no signs of meningitis.

Investigations

**Figure 2:**
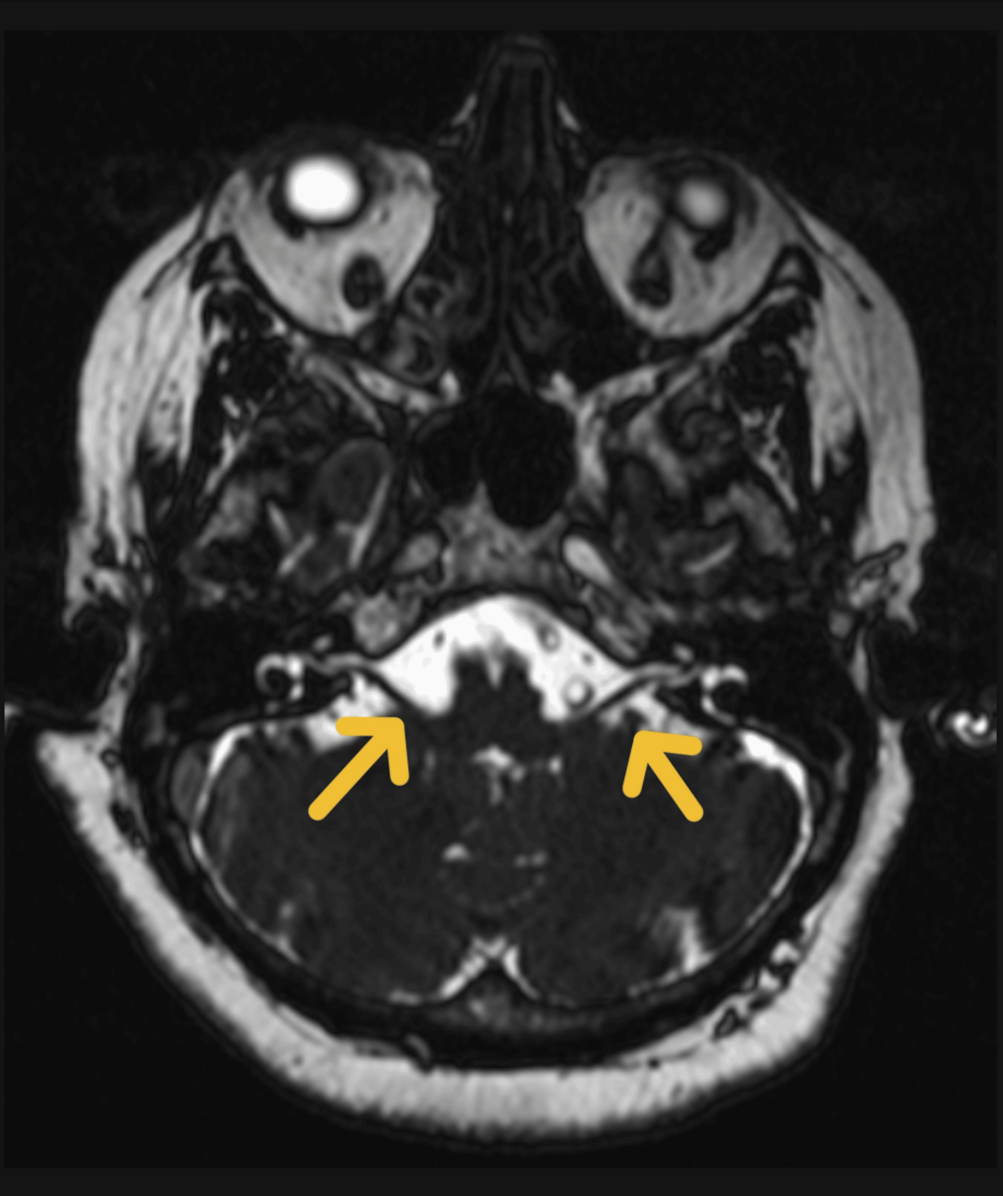
MRI of the brain (axial T2-weighted image) Axial T2-weighted MRI of the brain showing normal parenchymal architecture. Yellow arrows point to cranial nerve VII emerging from the pontomedullary junction. The nerves appear normal in course and caliber, with no evidence of enhancement and thickening.

Complete blood counts were within normal limits except for mild leucocytosis; detailed results are provided in Table 1. Renal function tests and electrolyte levels were within normal limits (Table 2). Liver function tests showed mild elevation in alkaline phosphatase (Table 3). The blood glucose profile revealed elevated postprandial glucose and HbA1c in the diabetic range, suggestive of previously undiagnosed diabetes, with dyslipidemia (Table 4). Viral markers were negative. Chest X-ray showed bilateral hilar prominence. Hence, suspecting sarcoidosis, serum angiotensin-converting enzyme levels and serum calcium levels were normal. MRI of the brain with contrast showed no evidence of stroke, demyelination, space-occupying lesion, or infection (Figure 2). A PET-CT scan was performed to rule out paraneoplastic manifestations and was reported as normal. To rule out Guillain-Barré Syndrome, nerve conduction studies (NCS) of all four limbs were done, which were normal. An ENT consultation was obtained, and no vesicles were found in the ear. An ophthalmology consultation was also obtained, which revealed no abnormalities. CSF analysis was not done, as the patient did not give consent.

**Table 1:** Complete blood count report Mild leucocytosis was noted; all other parameters were within normal limits. RBC: red blood cell; ESR: erythrocyte sedimentation rate

Complete Blood Count	Result	Reference Range
Hemoglobin	12.2g/dl	12-15 g/dl
Total leucocyte count	15,690 cells/ mm^3^	4000-11000 cells/ mm^3^
Differential count		
Polymorphs	58.7%	45-70%
Lymphocytes	35.5%	25-40%
Eosinophils	0.4%	1-6%
Monocytes	4.2%	2-10%
Basophils	0.3%	0-1%
RBC count	4.23 million cells/mm^3^	3.8-4.8 million cells/mm^3^
Platelet count	0.341 million cells/mm³	0.15–0.45 million cells/mm³
ESR (one hour)	14 mm/hr	4-19 mm/hr

**Table 2:** Renal function tests and serum electrolytes Serum creatinine, BUN, and other electrolytes were within normal limits.

Renal Function Tests And Serum Electrolytes	Result	Reference Range
Sodium	139mmol/L	135-145 mmol/L
Potassium	3.6 mmol/L	3.5 - 5.0 mol/L
Chloride	101mmol/L	98-107 mol/L
Bicarbonate	29 mmol/L	22-29 mmol/L
Creatinine	0.6 mg/dl	0.5-0.9 mg/dl
Blood urea nitrogen (BUN)	13 mg/dl	6-20 mg/dl
Calcium	9.1 mg/dl	8-11 g/dl

**Table 3:** Liver function tests Mild elevation in alkaline phosphatase was noted; other parameters were within normal limits. SGOT: Serum glutamic-oxaloacetic transaminase; SGPT: serum glutamic pyruvic transaminase

Liver Function Tests	Result	Reference range
SGOT	17 IU/L	<32 IU/L
SGPT	16 IU/L	<33 IU/L
Alkaline phosphatase	113 IU/L	35-104 IU/L
Total protein	7.5 g/dl	6.6-8.7 g/dl
Albumin	4.1 g/dl	3.9-4.9 g/dl
Globulin	3.4 g/dl	2.0-3.5 g/dl
Total bilirubin	0.50 mg/dl	<1.2 mg/dl

**Table 4:** Laboratory investigations Postprandial blood glucose elevation was noted, HbA1c was noted to be in the diabetic range, while other parameters were within normal limits. Serum ACE levels were normal. HbA1c: glycated hemoglobin; HDL: high-density lipoprotein; LDL: low-density lipoprotein; ACE: angiotensin converting enzyme

Test	Result	Reference Range
Fasting blood glucose	88 mg/dl	70-140 mg/dl
Two-hour postprandial blood glucose	310 mg/dl	80-140 mg/dl
HbA1c	6.9%	Normal: <5.7%; Prediabetic: 5.7-6.4%; Diabetic :>6.4%
Total cholesterol	218 mg/dl	0-200 mg/dl
Triglycerides	103 mg/dl	0-150 mg/dl
HDL cholesterol	68 mg/dl	85-60 mg/dl
LDL cholesterol	136 mg/dl	0-100 mg/dl
Serum ACE	15 microgram/ L	<40 microgram /L

As no alternative etiology was identified and the onset closely followed varicella infection, bilateral facial nerve palsy was most plausibly attributed to VZV-related post-infectious neurological complication. She was treated with oral corticosteroids, tab prednisolone 60 mg/day for 10 days, and physiotherapy, including transcutaneous electrical nerve stimulation (TENS), was also administered.

After discharge, the patient was on regular follow-up, she continued her physiotherapy, and steroids were tapered and stopped gradually over two weeks. The patient recovered completely after one month.

## Discussion

The VZV causes two distinct illnesses: chickenpox (varicella) and shingles (herpes zoster). Varicella occurs due to primary infection, and it is more common in childhood but can also affect adults. The incubation period ranges from 10 to 21 days [5]. It typically presents with a rash, which initially presents as small erythematous macules that progress to papules, vesicles, pustules, and subsequent central umbilication and crust formation over the body and face, low-grade fever, and malaise [6]. While often self-limiting, varicella can sometimes have complications such as secondary bacterial infections, otitis, pneumonia, hepatitis, necrotizing fasciitis, nephritis, arthritis, myocarditis, corneal lesions, and bleeding diathesis. Common central nervous system complications of varicella include encephalitis, meningitis, ventriculitis, and cerebellar ataxia; rarer manifestations include Guillain-Barré syndrome, meningoencephalitis, ventriculitis, optic neuritis, herpes zoster ophthalmicus, transverse myelitis, aseptic meningitis, post-herpetic neuralgia, delayed contralateral hemiparesis, peripheral motor neuropathy, cerebral vasculitis, Reye syndrome, and facial paralysis [7,8]. The latency period for neurological complications can vary from eight to 38 days. The proposed mechanisms for these neurological complications include direct neurological damage, immune-mediated damage, and vasculopathy [9]. Facial nerve palsy is a rare complication, and bilateral involvement is exceedingly uncommon, particularly in immunocompetent individuals. In our case, bilateral facial nerve palsy presented 30 days after the onset of chickenpox.

In our case, the patient presented with bilateral facial nerve palsy four weeks after the onset of chickenpox symptoms. She experienced slurred speech and difficulty sipping fluids. The inability to perform lip-dependent articulations is due to the loss of motor function in the lip muscles. Failure to purse the lips and seal the mouth resulted in leakage when drinking, highlighting the importance of the buccal and marginal mandibular branches in speech and swallowing. This impairment is more severe in cases of bilateral facial nerve palsy compared to unilateral palsy. Hence, extra attention should be given to bilateral cases due to the potential for feeding disabilities. 

The prognosis for patients with facial nerve palsy following varicella infection is usually good, with the majority of known cases experiencing complete facial nerve recovery within six months [10-12]. Previously reported cases have been treated with a combination of antiviral and steroid medications, either orally or intravenously, with mostly positive outcomes. In this case, as the patient had already received acyclovir, she was given oral prednisolone 60 mg per day, which was given for 10 days and was gradually tapered and stopped. She also underwent physiotherapy exercises, including TENS, and received education on eye protection measures. The patient was followed up regularly and reported complete recovery within four weeks.

In our patient, Ramsay Hunt syndrome was ruled out due to the absence of herpetic vesicular eruptions around the ear. Bell's palsy was unlikely given the preceding varicella infection and bilateral involvement. Sarcoidosis was excluded as serum calcium and ACE levels were normal. Lyme disease was ruled out based on the absence of travel history, and Guillain-Barré syndrome was ruled out as there was no limb weakness, areflexia, or sensory deficit, and NCS was normal. Meningitis and encephalitis were considered unlikely, as there were no signs of meningeal irritation or altered sensorium, and the MRI brain with contrast was normal.

A limitation of this case report is that the patient did not undergo CSF analysis and VZV IgM serology, which is a confirmatory test for chickenpox. However, chickenpox is typically considered a clinical diagnosis.

Given the preventable nature of varicella infection, vaccination remains the most effective measure to reduce disease burden and severity of complications. Two doses of vaccine are recommended, especially for healthcare professionals, immunocompromised individuals, children, international travelers, and military personnel, if not contraindicated [5]. In developing countries, vaccination plays a crucial role in preventing such infections and their associated complications.

## Conclusions

Although chickenpox infection is mild for most individuals, it can occasionally present with serious complications. Bilateral facial nerve palsy due to primary VZV infection is extremely rare, particularly in immunocompetent individuals. Early recognition and timely treatment with corticosteroids and physiotherapy can result in complete recovery. This case highlights the importance of varicella vaccination in reducing the risk and severity of complications.
